# Medicolegal consequences of altered COVID-19 vaccine administration

**DOI:** 10.1177/01410768211005112

**Published:** 2021-04-06

**Authors:** Gordon Muir, Jeremy Crowther

**Affiliations:** 1 King’s College Hospital, London SE5 9RS, UK; 2 9 Gough Square Chambers, London, UK

While there have been recent calls for the introduction of laws to indemnify doctors and NHS trusts who rationed care during the pandemic, other issues relating to vaccination of healthcare workers may be of more direct relevance to UK doctors and NHS managers.

Unlike the general population, healthcare workers do not have the option to self-isolate or work from home. NHS frontline staff were around four times more likely to contract COVID-19 during the first wave than the general public, and their families twice as likely. Nurses were twice as likely to die of COVID-19 compared to their peers.

While problems with personal protective equipment were widespread and probably causative in some cases, that was a general supply problem.

There has been debate as to the effectiveness of a single dose of the Pfizer/Biontech vaccine which has been given to most NHS frontline staff. It now seems that efficacy tails off from the fourth week after a single dose, so many NHS staff remain at risk of infection.

Most of these workers gave consent to be vaccinated with the Pfizer vaccine expecting a second dose at three weeks, only to have the posology changed to an unlicensed use (without their consent) after the event.

The NHS has thus put frontline staff at increased risk of disease and death (however small that is shown to be) by refusing them immunisation in line with almost all external advice, and by extending the second vaccination to 12 weeks or more, instead of the three weeks recommended by the manufacturer. Some NHS staff will have contracted COVID-19 in the window between the recommended second dose at three weeks and their eventual second dose. Many would not have done so if vaccinated to the manufacturer’s recommendations. Some are likely to die. On the balance of probability, vaccination according to licence would have reduced these risks.

Affected staff, or their dependants, may well have an actionable case against the staff vaccinating them or their employers, for they were plainly being exposed to a preventable foreseeable risk.

This will impact two groups of doctors (apart from those being partially immunised who may get sick or die for want of immunity).

First, those administering or prescribing the vaccine must be aware that they have done so as an unlicensed drug in direct conflict with the advice given by the manufacturer, the World Health Organization and the British Medical Association. This is essentially a trial which has not been passed by an ethics committee and to which patients did not consent. Doctors prescribing unlicensed or trial drugs should usually only do so in the context of enhanced informed consent. Moreover, it is clear that the available vaccines provide different degrees of immunity after one dose. Should patients not have been told of the relative efficacy of all available vaccines, and given the option to wait for immunisation with their preferred product?^1^ Many healthcare workers we have spoken to would not have had the Pfizer vaccine had they known the second dose would be delayed by months.

Chief executives and board members of NHS trusts have, like all employers, a duty of care to their employees. If staff are on one hand being asked to work extra shifts on COVID-19 wards and having annual leave cancelled, it does seem perverse that their employers will not do everything in their power to protect them in circumstances where the risk could be mitigated by a second vaccination. However, we are interested in the law, not the moral aspects of this.

The greater the risk and gravity of consequence, the greater care that should be taken by the employer^2^: why then the point blank refusal of the Department of Health to allow healthcare workers the second vaccine on time in any circumstance?

Trust executives should have made it clear that they did not and do not approve of this risk to their staff. The ex-health minister Lord Warner said in a letter to *The Times* that this would have been a resignation issue for him. We wonder why it did not seem to be so for senior NHS executives who have a primary duty of care to their staff, not the general herd immunity.

It will be interesting to see how many cases are brought in the coming months, and what the defence of individuals and NHS trusts will be.
